# Macroscale coupling between structural and effective connectivity in the mouse brain

**DOI:** 10.1038/s41598-024-51613-7

**Published:** 2024-02-07

**Authors:** Danilo Benozzo, Giorgia Baron, Ludovico Coletta, Alessandro Chiuso, Alessandro Gozzi, Alessandra Bertoldo

**Affiliations:** 1 Department of Information Engineering, University of Padova, Padua, Italy; 2 Functional Neuroimaging Laboratory, Center for Neuroscience and Cognitive Systems @ UniTn, Istituto Italiano di Tecnologia, Rovereto, Italy; 3 Padova Neuroscience Center (PNC), Padua, Italy

**Keywords:** Dynamical systems, Network models

## Abstract

Exploring how the emergent functional connectivity (FC) relates to the underlying anatomy (structural connectivity, SC) is one of the major goals of modern neuroscience. At the macroscale level, no one-to-one correspondence between structural and functional links seems to exist. And we posit that to better understand their coupling, two key aspects should be considered: the directionality of the structural connectome and limitations in explaining networks functions through an undirected measure such as FC. Here, we employed an accurate directed SC of the mouse brain acquired through viral tracers and compared it with single-subject effective connectivity (EC) matrices derived from a dynamic causal model (DCM) applied to whole-brain resting-state fMRI data. We analyzed how SC deviates from EC and quantified their respective couplings by conditioning on the strongest SC links and EC links. We found that when conditioning on the strongest EC links, the obtained coupling follows the unimodal-transmodal functional hierarchy. Whereas the reverse is not true, as there are strong SC links within high-order cortical areas with no corresponding strong EC links. This mismatch is even more clear across networks; only within sensory motor networks did we observe connections that align in terms of both effective and structural strength.

## Introduction

How structural connectivity (SC) is coupled with functional brain properties remains an open question for modern neuroscience^1^. The investigation of this problem is highly influenced by the chosen scale of analysis. Even at the micro scale, where detailed microcircuit structures can be coupled with biophysical models, there is still a challenge in replicating empirical functional properties^2^. This challenge becomes more prominent at larger scales, where bottom-up models become increasingly complex, making them difficult to handle. As a result, top-down models emerge as the primary alternative. Focusing on the macroscale case, which is the target of this work, the top-down models currently available are of two main types^3,4^: brain network model (BNM)^5^ and dynamic causal modeling (DCM)^6^. In both cases, SC is often used to constrain the interactions across brain units. In BNM this is a strong a priori assumption, as single unit brain dynamics are placed on top of a structural matrix—to mitigate this assumption a communication-inspired BMN has recently been developed^7^. Within the DCM framework, even if in principle there is no need for structurally informed prior, several DCM variants have been developed that incorporates structural information, thus reducing the dimensionality of the model and meeting the assumption that a functionally effective link implies an underlying structural link^8,9^.

However, given that from micro to macro scale functional connectivity (FC) deviates more and more from SC^10^, there is no clear consensus that a structurally informed prior is always beneficial. A possible explanation for this phenomenon could be that the contribution of emergent network properties may outweigh the importance of a single brain region, thus conflicting with the pairwise nature of the structural information^11,12^. Further works also investigated the heterogeneity in the structure–function coupling across neocortical areas, and found that the overlap between FC and SC is maximal in primary sensory and motor regions, and it gradually decreases toward a global minimum in transmodal brain areas^13–16^. This latter result is in line with previous work showing that a sensory fugal gradient in the spatial organization of FC represents one fundamental axis capturing the intrinsic architecture of the cortex^17^.

A more recent line of research has focused on how SC relates with the dynamics of brain activity, thus going beyond the static steady-state nature of FC. For example, Liu et al.^18^ studied how the coupling between SC and dynamic FC evolves in time, while Gu et al.^19^ tried to relate functional modular flexibility with structure by means of a measure of controllability. Lastly, Avena-Koenigsberger et al.^20^ provided a comprehensive overview of key aspects of communication dynamics and their link with topological properties of SC. This brought us to the use of the effective connectivity (EC) matrix as a means to analyze brain dynamics. Traditionally, EC refers to the causal influence that each element of a system exerts on the dynamics of the other elements^21^. EC is not directly measured, whereas it is instead encoded as a parameter of the state-space DCM model and estimated through model inversion. For many years, the computational complexity of model inversion prevented the application of DCM to whole-brain data and limited its use to few brain regions. However, recently proposed solutions have enabled scaling DCM up to the whole-brain level, thus paving the way for our work^22–24^.

Here, our interest lies in exploring the relationship between SC and EC. However, due to the lack of reliable information regarding directed anatomical connectivity of the human brain, it is difficult to validate EC models in humans. Indeed, human SC is commonly reconstructed from non-invasive and easily available diffusion-weighted MRI which is not precise in tract reconstruction^25,26^. Furthermore, this method yields a symmetric connectivity matrix that contrasts with the asymmetric nature of EC^27^. As an alternative, viral tracer techniques on animal models provide an accurate reconstruction of monosynaptic axonal path and are considered as the gold standard for mapping the structural connectome^28,29^. This is exemplified by the mesoscale connectome of mouse brain mapped through directional viral traces^30^, offering a better resolution than MRI can achieve in primates and humans. Furthermore, also concerning the functional data, the mouse model represents a valid choice. Robust protocols have been developed for acquiring resting-state fMRI data, ensuring the preservation of critical functional and dynamic properties inherent to resting-state BOLD signals^29,31,32^. These resources represent an ideal framework for studying the coupling between SC and EC due to its directed organization.

In the present work, we studied the relation between effective connectivity and directed structural connectivity at the whole-brain level in the mouse brain. EC was computed subject-wise on a dataset of resting-state fMRI BOLD signals recorded from 20 anesthetized mice by means of sparse DCM^24^. Global directed SC was inferred from a directed weighted voxel-wise model of the mouse brain^33^ obtained through viral tracings. We showed that the strongest structural links are associated with the strongest EC links, and vice-versa. However, a more detailed analysis at the node level revealed that the coupling strength changes in a network dependent fashion based on whether the link selection was based on the strongest EC or SC links. Moreover, we specifically examined the impact of the hypothesis that an effective link necessitates a structural counterpart on model inference and its generative capabilities at the macroscale level. Our results emphasize the need to differentiate between within and across network links when evaluating the impact of a structural constraint at various levels of stringency. A too strict structural constraint negatively affected the model fit, as it does not permit effective links to deviate from it sufficiently. This is particularly relevant for between-network links and within high-order cortical areas; however, effective links within unimodal motor-sensory areas appear to align with the structural pathway regardless of any restraint.

## Materials and methods

### Data collection and preprocessing

A dataset of n = 20 adult male C57BI6/J mice were previously acquired at the Italian Institute of Technology (IIT) laboratory (Italy). All in vivo experiments were conducted in accordance with the Italian law (DL 2006/2014, EU 63/2010, Ministero della Sanità, Roma) and the recommendations in the Guide for the Care and Use of Laboratory Animals of the National Institutes of Health. Animal research protocols were reviewed and consented by the animal care committee of the Italian Institute of Technology and Italian Ministry of Health. Animal preparation, image data acquisition and image data preprocessing for rsfMRI data have been described in greater detail elsewhere^32^. Briefly, rsfMRI data were acquired on a 7.0-T scanner (Bruker BioSpin, Ettlingen) equipped with BGA-9 gradient set, using a 72-mm birdcage transmit coil, and a four-channel solenoid coil for signal reception. Single-shot BOLD echo planar imaging time series were acquired using an echo planar imaging (EPI) sequence with the following parameters: repetition time/echo time, 1000/15 ms; flip angle, 30°; matrix, 100 × 100; field of view, 2.3 × 2.3 cm^2^; 18 coronal slices; slice thickness, 0.60 mm; 1920 volumes. During the acquisition of functional data mice were anesthetized with 0.75% halothane.

Regarding image preprocessing as described in Gutierrez-Barragan et al.^34^, timeseries were despiked, motion corrected, skull stripped and spatially registered to an in-house EPI-based mouse brain template. Denoising and motion correction strategies involved the regression of mean ventricular signal plus 6 motion parameters^35^. The resulting timeseries were then band-pass filtered (0.01–0.1 Hz band). After preprocessing, mean regional time-series were extracted for 74 (37 + 37) regions of interest (ROIs) derived from a predefined anatomical parcellation of the Allen Brain Institute (ABI)^30,36^.

### Cortical network partitions

We partitioned the functional cortical networks into the lateral cortical network (LCN)^37^, the default mode posterolateral network (DMNpost), the default mode midline network (DMNmid) and the salience (SAL)^34^. In particular, LCN includes: primary and secondary motor, and primary and supplementary somatosensory areas. DMNpost contains: gustatory, posterior parietal association, temporal association and visceral areas. DMNmid contains: anterior cingulate (dorsal and ventral), prelimbic, infralimbic, orbital and retrosplenial (agranular, ventral and dorsal) areas. SAL refers to the agranular insula areas (dorsal, posterior and ventral parts).

### Sparse-DCM

The effective connectivity matrix was estimated at the single subject level using the method described in Prando et al.^24^, called sparse-DCM (Dynamic Causal Modeling). In line with the DCM framework^6^, sparse-DCM is a state-space model where the state *x(t)* satisfies a set of linear differential equations representing the coupling among neural components, and the output model maps the neuronal activity to the measured BOLD signal *y(t)* through the hemodynamic response function (HRF):

1 $$\frac{dx}{dt}=Ax\left(t\right)+\nu \left(t\right)$$

2 $$y\left(t\right)=h\left(x\left(t\right);{\theta }_{h}\right)+e\left(t\right)$$

with* A* representing the effective connectivity matrix, *h(.)* the hemodynamic response that is modeled by the biophysically inspired Balloon–Windkessel model^6^ and *θ*_*h*_ its parameters. *v(t)* denotes the stochastic intrinsic brain fluctuations and *e(t)* the observation noise, both are Gaussian variables with zero mean and diagonal covariance matrices *σ*^*2*^*I*_*n*_ (*I*_*n*_ the identity matrix of size *n*) and *R* = diag(*λ*_*1*_, *λ*_*2*_, …, *λ*_*n*_), respectively.

To address the computational burden of model inversion when dealing with whole brain data, in Prando et al.^24^ the authors proposed a discretization and linearization of Eqs. (1, 2) as well as a sparsity-inducing prion on the EC matrix, i.e. *A* in Eq. (1). This was motivated by the low temporal resolution of fMRI data, which usually ranges from 0.5 to 3 s, and the idea that the hemodynamic response *h(.)* can be modeled as a Finite Impulse Response (FIR) model with input the neuronal state and output the BOLD signal. In our study, to ensure that the length of the input response was large enough to model relevant temporal dependencies, we set the hemodynamic length to 18 samples with a sampling time 1 s (TR). For each brain parcel *i*, a finite impulse response *h*_*i*_ ~ *N(μ*_*h*_*, Σ*_*h*_*)* was assigned by deriving *μ*_*h*_ and* Σ*_*h*_ through a Monte-Carlo sampling of typical responses generated by the non-linear Balloon-Windkessel model (10,000 samples).

Since we worked with mouse data, we did not utilize the parameters *θ*_*h*_ as proposed in Friston et al.^6^, which were designed for human data. Instead, we employed the blind deconvolution approach outlined in Wu et al.^38^ to obtain an HRF estimate at the subject level. Subsequently, we fitted a Balloon–Windkessel model to derive optimal θ^*^_h_ values that better replicated the estimated HRF. Averaging these values across subjects, we obtained a mouse-based prior vector θ^*^_h_ = *[κ* = 0.37*, γ* = 0.20*, τ* = 2.75*, α* = 0.50*, ρ* = 0.51*]*, refer to Friston et al.^6^ for their biophysical interpretation.

The sparsity-inducing prior on the EC estimation was formulated to reduce as much as possible spurious couplings. In particular, each element *a*_*i*_ of matrix *A* was assumed to be a Gaussian variable with zero mean and *γ*_*i*_ variance. The hyperparameter *γ* = *[γ*_*1*_*, γ*_*2*_*, …, γ*_*nxn*_*]* was estimated through marginal likelihood maximization. Under generic conditions, the maximum likelihood estimate of some *γ*_*i*_-s will be zero such that the Gaussian posterior distribution of their corresponding *a*_*i*_ is concentrated around zero thus producing a zero MAP estimate. In sparse-DCM, model inversion and parameter optimization are performed by an expectation–maximization (EM) algorithm.

To evaluate the influence of a structurally-informed constraint on the estimation of EC, a portion of our analysis involved integrating this constraint into the sparse-DCM method. This was achieved by imposing the requirement that certain EC entries be set to zero during the inference process (note that, unless otherwise explicitly mentioned, the inference was performed without any constraints).

This subset was chosen from the structural connectivity (SC) matrix and consisted of links with a strength below a certain threshold. To get a better understanding of the effect of this constraint, three thresholds were tested, corresponding to the 60th, 40th and 20th percentiles of the SC entry distribution. The inclusion of structurally informed constraints in the estimation process of sparse-DCM, thereby providing a generative model in which these constraints are embedded, represents a powerful tool for testing mechanistic hypotheses^39^. This is a distinct approach compared to those employing SC as informative priors to facilitate model identification, which can be seen for example in Stephan et al. and Sokolov et al.^8,9^.

### Structural connectivity

The directed structural matrix was inferred from a directed weighted voxel-wise model of the mouse brain^33^ obtained through multiple viral microinjection experiments (Allen Mouse Brain Connectivity Atlas) and re-parceled into the same number of regions used for the rsfMRI data^29^.

### Structure-effective coupling

The structure-effective coupling was studied both at the global level and at the node level using the EC matrices obtained with the non-structurally-informed DCM, unless stated otherwise. In the first case, we considered how the strength of all EC links distributes according to the structural connectome. In particular, we binarized the log-SC with different thresholds (from 10 to 90%, step 20) and used it to mask the EC. We refer to this as the global SC–EC coupling. Practically, we looked at the mean absolute EC strength of links both with and without a structural connection, MA(EC_strlink_) and MA(EC_nostrlink_) respectively, and normalized each mean with the global mean absolute EC, MA(EC):

3 $$globalSC\to ECcoupling=\frac{MA\left({EC}_{strlink}\right)}{MA\left(EC\right)}=\frac{MA\left(EC\cap {SC}_{bin}^{th}\right)}{MA\left(EC\right)}$$

where ${SC}_{bin}^{th}$ denotes a binarization of the SC matrix, with entries set to 1 if they are above the *th* percentile of its entry distribution, and 0 otherwise. The same procedure was then repeated by reversing the role of the two measures, namely, thresholding the effective and evaluating the strength of the structure, i.e. the global EC–SC coupling:

4 $$globalEC\to SCcoupling=\frac{MA\left({SC}_{efflink}\right)}{MA\left(SC\right)}=\frac{MA\left(SC\cap {EC}_{bin}^{th}\right)}{MA\left(SC\right)}$$

**Figure 1:**
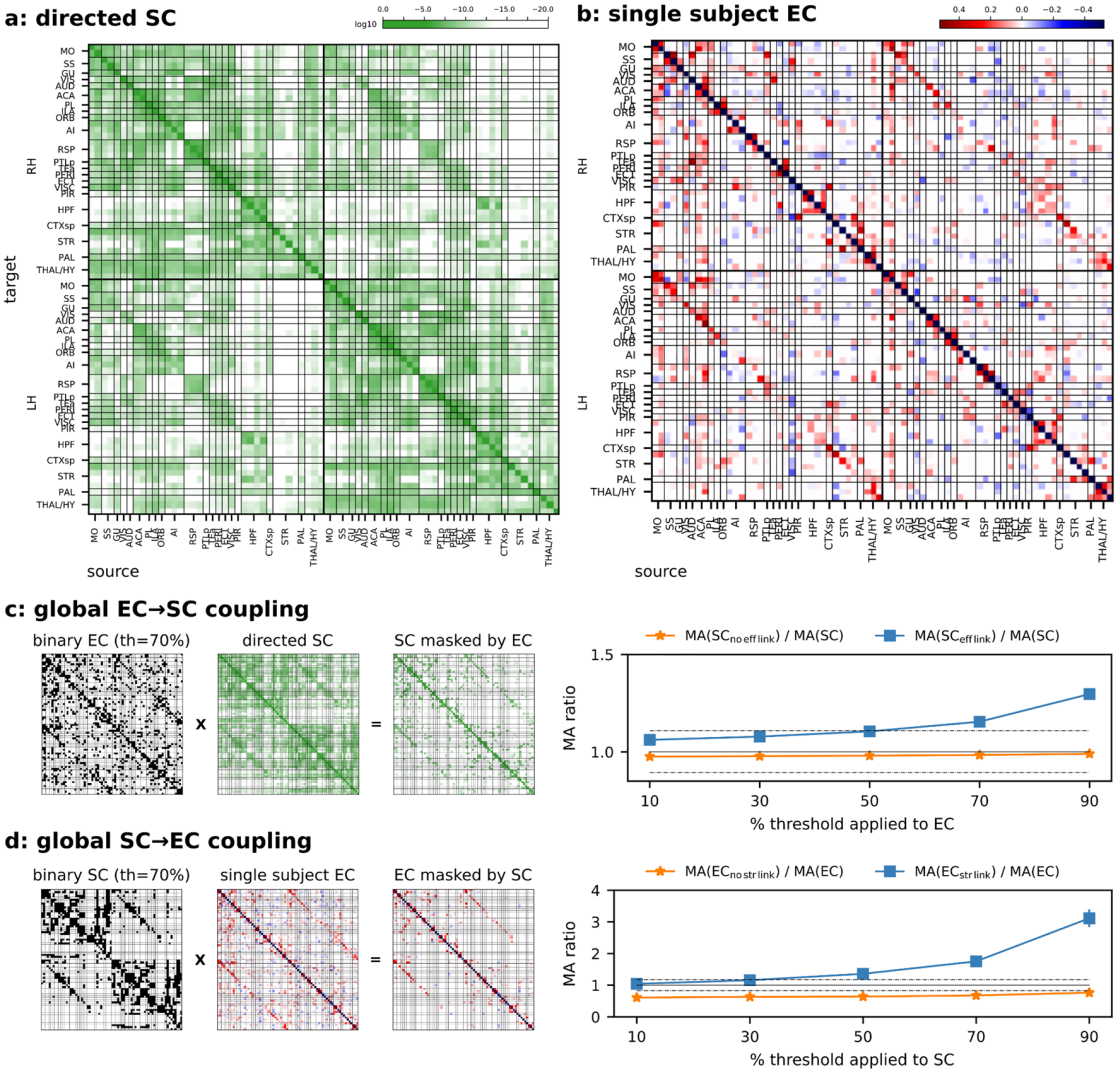
(**a**) The directed log-structural connectivity at the macroscale, obtained by a weighted voxel-wise model applied on the mouse axonal connectome. (**b**) Effective connectivity matrix of a representative subject (note that no structurally-informed constraint was imposed during this analysis), excitatory links are in red and inhibitory links in blues. In both matrices, target regions are on the y axis, and source regions on the x axis. Regions are grouped by hemisphere and sorted from cortical to subcortical areas. (**c**) EC → SC coupling: SC was masked by each single subject binary EC (given a certain threshold) and computed the related MA (mean absolute) ratios both for zero (orange line) and nonzero (blue line) mask entries. (**d**) SC → EC coupling, similarly to panel (**c**) with the mask derived from SC and applied on EC (for each subject and binarization thresholds). In both panels (**c**, **d**) an MA ratio greater than one on non-zero mask entries (blue lines) implies on average a high SC (EC) strength on nodes with high EC (SC) strength. As means of comparison, MA ratios computed within and across hemispheres are shown as dashed lines (> 1 within, and < 1 across).

As means of comparison, in Fig. 1c,d the MA ratios computed within and across hemispheres are also presented (indicated by dashed lines). These ratios were computed in line with Eqs. (3,4) selecting only entries within and across hemispheres in the EC and SC matrices, respectively.

To study the relation at the node level, we computed the coupling at each node for both incoming and outgoing links, i.e. by considering separately the rows and columns of the connectivity matrices, respectively. To this end, the node-wise coupling between structural and effective links was computed as the Spearman rank correlation between the top *k* entries of the effective and the related structural ones: we refer to this as the EC–SC coupling. Similarly, for each node the SC–EC coupling was computed as the Spearman rank between the top *k* entries of its structural vector and the corresponding effective entries. We will report results with *k* = 15, that for our data corresponds to the 20% of ROIs in the adopted parcelization (in Supplementary Materials, the coupling is shown under different thresholds, and statistical significance tested through random permutation test).

To quantify the deviation of the strongest SC links of a given node from its strongest EC links, as well as vice-versa, we computed the *only SC* and *only EC* rates. The *only SC* rate is defined as the number of *only SC* over the total amount of considered links (*only SC* + *overlap*):

5 $$onlySCrate=\frac{onlySC}{onlySC+overlap}$$

where for a given node *only SC* is the number of structural links which do not correspond to an effective links and the *overlap* is the number of links that are both structural and effective.

Similarly, for the *only EC* rate we define:

6 $$onlyECrate=\frac{onlyEC}{onlyEC+overlap}$$

When the rate equals 0, there are no lonely SC (or EC) connections; if it equals 0.5, then lonely and overlapped connections are equally distributed; if it equals 1, there is no overlap between effective and structural links. As in the previous analysis, the strongest links were selected with *k* = 15.

In this work, we considered the strength of each EC connection independently from its sign. Thus, when referring to EC, we mean the absolute value of the actual state-space matrix of sparse-DCM.

### Goodness of fit metrics

After fitting a DCM model to each empirical single-subject rsfMRI recordings, we generated 100 realizations of synthetic rsfMRI signals (by using the MATLAB function *lsim*). To assess the goodness of fit between the empirical and simulated data, we calculated the correlation between their functional connectivity, i.e. the correlation between the triangular part of the empirical FC and simulated FC, and the similarity between the distributions of their dynamic FCs. In detail, to capture time-dependent properties of the data, the dynamic FC was computed using a sliding window of 50 s (with 25 s step), and we used the Kolmogorov–Smirnov distance between the triangular part of the empirical dFC and simulated dFC as dissimilarity metric^40^.

## Results

On each mouse rs-fMRI scan, we applied sparse-DCM and obtained the related effective connectivity matrix. We used the model and implementation proposed in Prando et al.^24^. The computational time needed per subject was in the order of tens of hours on a computer cluster that had 12 Intel Xeon 3.20 GHz CPUs and 110 Gb of RAM allocated. Figure 1b shows the effective connectivity of a representative subject and, in Fig. 1a, the global directed structural matrix.

Firstly, we looked at the coupling between the two matrices from a global perspective. We thus considered the mean absolute (MA) strength of the structural connectivity entries that are (and are not) associated with an effective link under different binarization thresholds applied on EC, and normalized with respect to the MA of the whole SC matrix: MA ratio (see Fig. 1c, the left side of the panel summarizes the procedure and on the right side MA ratio is reported for different thresholds). We refer to this as EC–SC coupling, since a binary EC mask was constructed to select the strongest links and then applied to the SC matrix, see Eq. (4).

The same analysis was repeated by reversing the role of the two measures, i.e. thresholding the structural and evaluating the strength of the effective entries (see Fig. 1d global SC–EC coupling, and Eq. (3)). In both cases, the increase in the mean absolute ratio with an increasing binarization threshold indicates a higher mean structural (effective) strength among nodes with the strongest effective (structural) connections. Table 1 displays the mean Spearman rank correlation, calculated using the same methodology as the MA ratio. The trends observed are consistent: when a connection is present, the mean correlation increases with the threshold, and it remains statistically significant for each subject. The only exception is the EC–SC coupling, which exhibits a lower correlation at 90% compared to 70%.

**Table 1:** Mean Spearman rank correlation (averaged z-values across subjects), under different binarization thresholds. Similarly to Fig. 1, in the EC → SC coupling, the SC was masked by each single subject binary EC (given a certain threshold), while for the SC → EC coupling the mask was derived from SC and applied on EC (for each subject and binarization thresholds).

%th	Global EC → SC coupling		Global SC → EC coupling	
	$\rho \left( {EC_{eff\;link} ,SC_{eff\;link} } \right)$	$\rho \left( {EC_{no\;eff\;link} ,SC_{no\;eff\;link} } \right)$	$\rho \left( {SC_{str\;link} ,EC_{str\;link} } \right)$	$\rho \left( {SC_{no\;str\;link} ,EC_{no\;str\;link} } \right)$
10	0.270*	0.009	0.172*	0.003
30	0.304*	0.029	0.195*	0.011
50	0.348*	0.044	0.220*	0.011
70	0.414*	0.064	0.265*	0.027
90	0.370*	0.102*	0.352*	0.066

*Significance in all subjects (Bonferroni corrected by the number of thresholds and link/nolink).

To better characterize the relationship between structural and effective connections, we quantified their coupling at the node level. The coupling was computed as the Spearman rank correlation for both incoming and outgoing links. Since both EC and SC are sparse matrices (the former by construction and the latter having sparsity bounded between 13 and 36% as reported in^30^), we computed their coupling by conditioning only on links of significant strength, discarding null or quasi-zero strength connections. Specifically, we computed the EC–SC coupling when conditioning on the effective links, and the SC–EC coupling when conditioning on the structural links.

**Figure 2:**
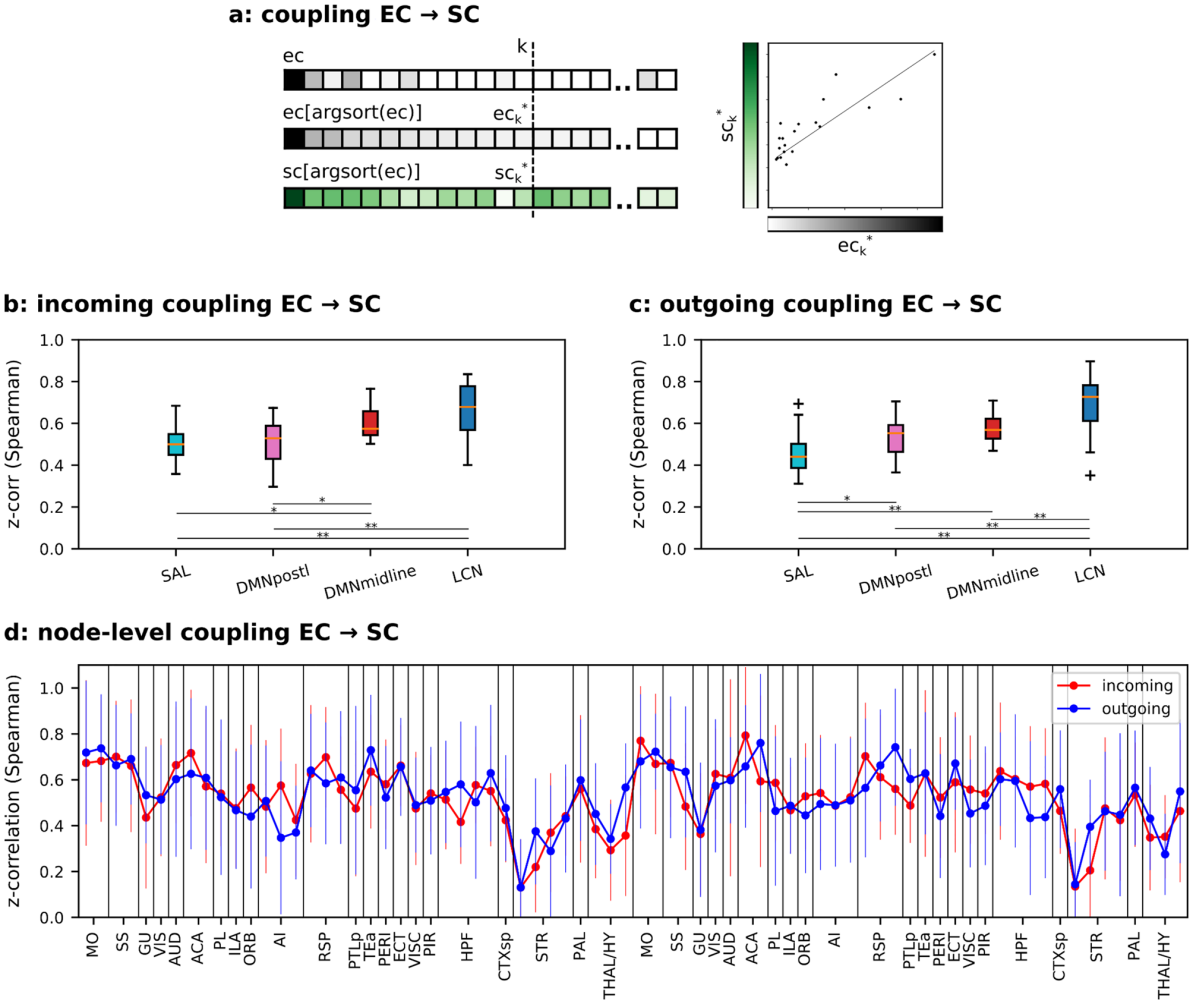
EC–SC coupling. (**a**) Explanatory cartoon of the EC–SC coupling computation for a given vector of links referred to the incoming or outgoing EC entries of a node (gray scale): the coupling is computed as the Spearman rank correlation between the strongest *k* EC entries and the related SC ones (green scale); argsort() gives the indices that would sort the vector. (**b**) Coupling across subjects computed on the incoming links and results grouped by functional networks with k = 15. (**c**) Similar to panel (**b**) on outgoing links. *p < 0.05, **p < 0.01 and ***p < 0.001, ANOVA test with Tukey’s multiple comparison test. (**d**) EC–SC coupling at the node-level. No significant differences between incoming and outgoing node couplings have been detected. Paired t-test, Benjamini/Hochberg multiple testing correction.

**Figure 3:**
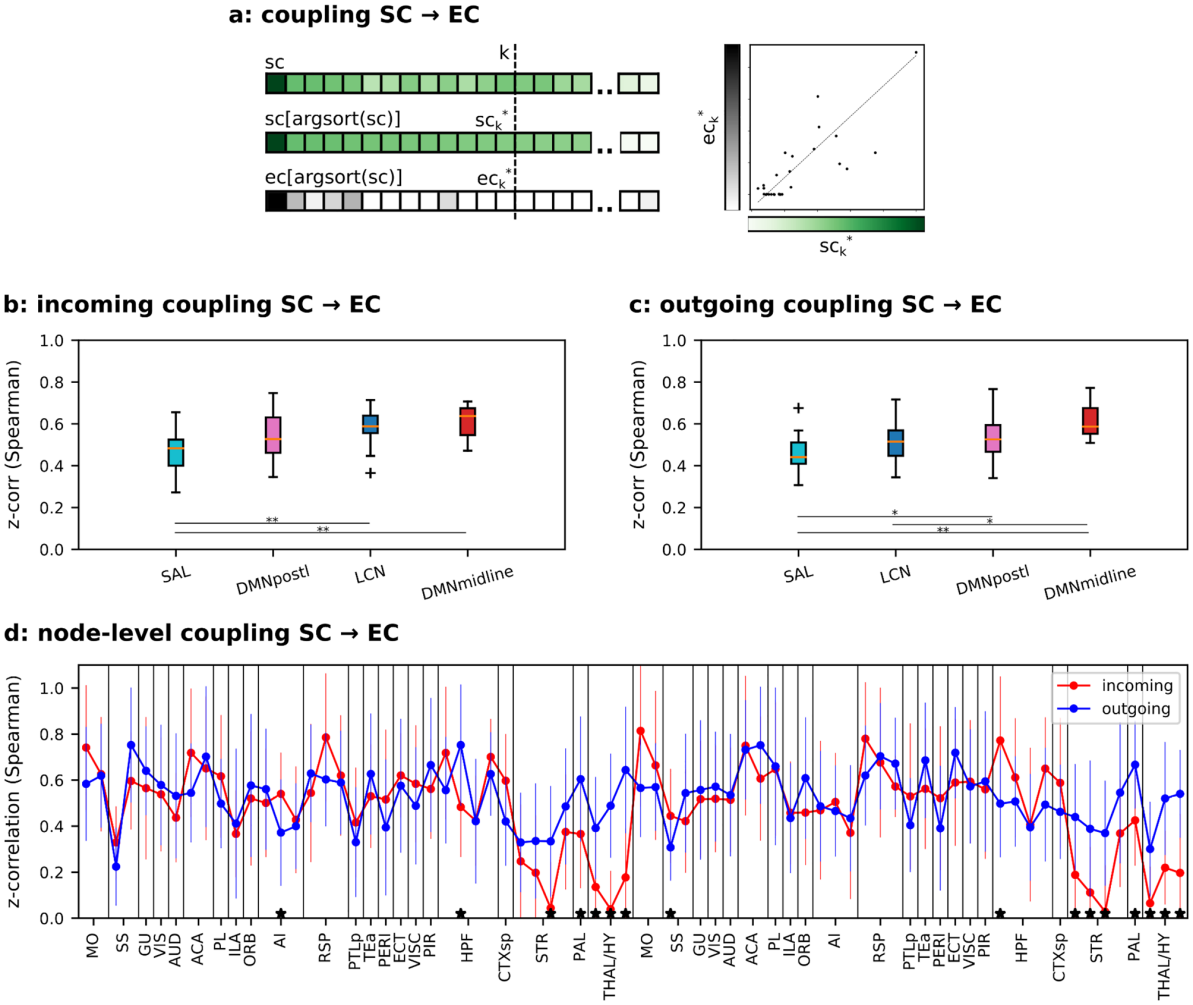
SC–EC coupling. (**a**) Explanatory cartoon of the SC–EC coupling computation for a given vector of links referred to the incoming or outgoing SC entries of a node (green scale): the coupling is computed as the Spearman rank correlation between the strongest *k* SC entries and the related EC ones (gray scale). (**b**) Coupling across subjects computed on the incoming links and results grouped by functional networks with k = 15. (**c**) Similar to panel (**b**) on outgoing links. *p < 0.05, **p < 0.01 and ***p < 0.001, ANOVA test with Tukey’s multiple comparison test. (**d**) SC–EC coupling at the node-level. *p < 0.05, paired t-test, Benjamini/Hochberg multiple testing correction.

The EC–SC coupling quantifies the correlation between a node’s most influential effective connections and its corresponding structural strengths. In other words, it evaluates whether the ordered node-wise effective vector is coherently supported by its structural vector (see Fig. 2a). On the other hand, the SC–EC coupling measures whether the strongest structural links of that node are supported by its respective effective links. In other words, we tested if the effective strength follows the anatomical strength (see Fig. 3a).

Figure 2 shows the EC–SC coupling, where panels b and c contain z-Spearman correlations across subjects with cortical nodes grouped by functional network for incoming and outgoing links respectively, and in panel d at the single node-level. Analyzing nodes grouped by functional network and sorted by coupling strength revealed a unimodal-transmodal hierarchy as previously described for the SC^29^. Indeed, transmodal networks, such as SAL and DMN postero-lateral, were found to be less coupled coupled with their underlying structural links, whereas EC links from unimodal motor and somatosensory areas (LCN) displayed higher levels of coupling. Importantly, no significant differences were detected between incoming and outgoing links.

Having reproduced a well-known functional hierarchy even without exploiting structural information when estimating EC reassured us on the validity of our model and prompted us to investigate the SC–EC coupling.

To this aim, the strongest SC links were selected for each node and correlated with the corresponding EC links (Fig. 3a). This is in line with the hypothesis that an effective link requires the presence of a structural link and it aims to test how the two measures are coupled.

Similarly to Fig. 2, Fig. 3 shows the coupling distribution across mice, with nodes grouped by their own functional network for incoming and outgoing links, respectively, in panel b and c. Interestingly, we saw that SC–EC coupling did not obey the previously observed functional hierarchy of EC–SC coupling. The SAL network exhibited the weakest coupling, yet for DMN and LCN there was not a clear ordering. Furthermore, in contrast to the previous case, the node-level coupling (Fig. 3d) showed a significant decrease in the incoming links of subcortical regions—striatum, pallidum and thalamus.

To elucidate the consequences of conditioning the coupling on the strongest EC or SC links, we conducted a comparison of the node-level incoming EC–SC and SC–EC couplings, as illustrated in Fig. S3a. This analysis confirmed the significant reduction of incoming SC–EC coupling for subcortical regions and unveiled a notably lower SC–EC coupling in the primary somatosensory area (SSp) compared to the EC–SC coupling. A similar reduction was evident when examining the outgoing coupling, as depicted in Fig. S3b. This reduction is likely associated with the disparity in ipsilateral and contralateral connections, both incoming and outgoing, between the profiles of structural and effective connectivity of SSp (for more details, refer to the legend of Fig. S3).

These discrepancies between EC–SC and SC–EC couplings motivated further investigation to better comprehend how the set of strongest SC links differs from the set of strongest EC links. To this end, we firstly computed the overlap rate between the *k*-th strongest SC and EC links (note that this ratio tends to 1 with *k* getting closer to the network size, 74 in our data). Figure S4 shows the overlap ratio per network across different values of *k* (from 15, i.e. 20% of the nodes, to 55, i.e. 74%, step 10). On average, with *k* = 15 the ratio is 0.41 for incoming links, and 0.42 for outgoing links. The overlap ratio reaches average values around 0.75 with *k* = 55.

This observation led us to focus on the strongest SC and EC links that do not overlap with each other. In particular, the strongest SC links that do not correspond to a high effective connection, as these are the root of the inconsistencies between couplings.

**Figure 4:**
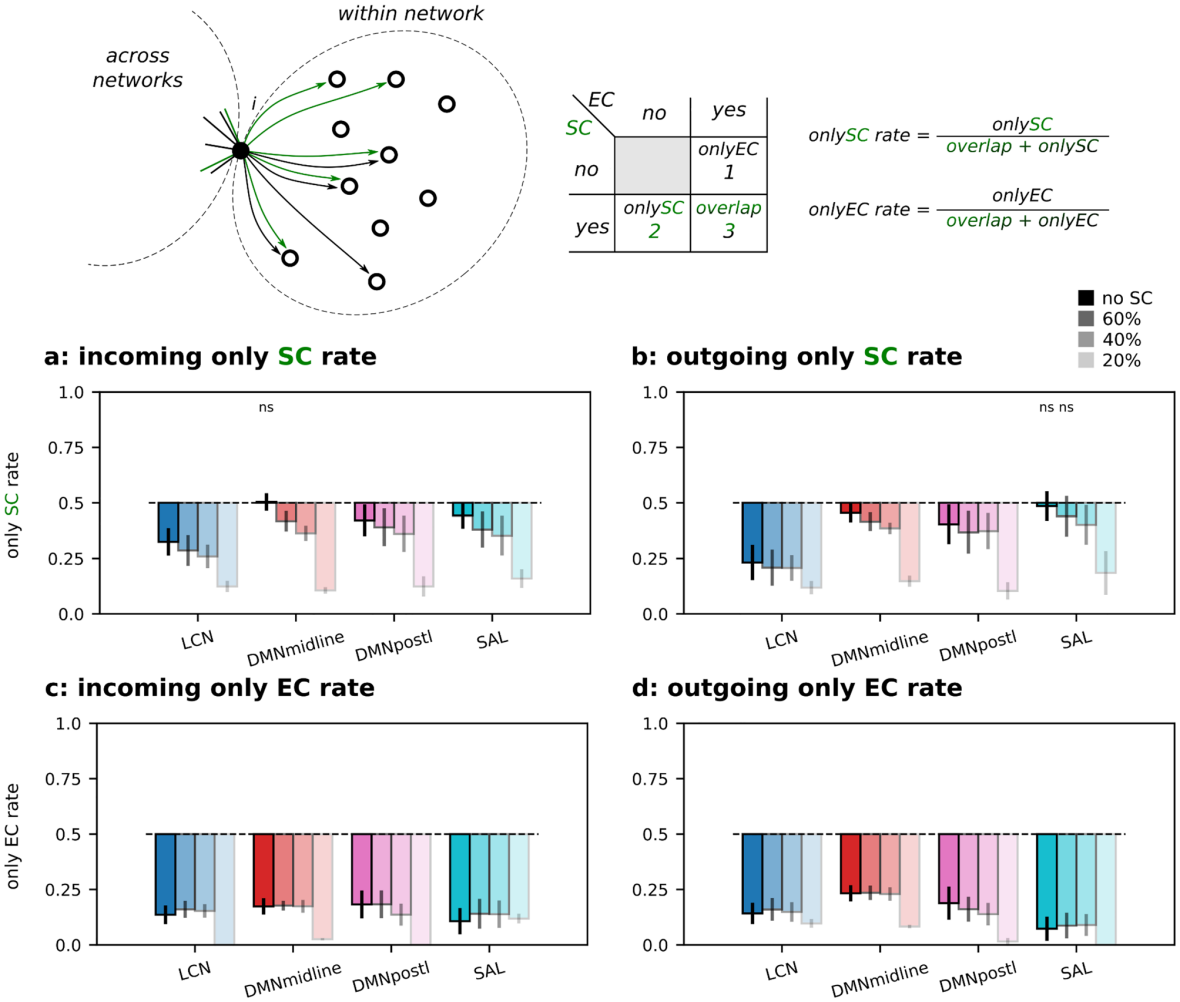
*Only SC* and *only EC* rates within networks. Explanatory cartoon of the *only SC* and *EC* rates computed on the within-network outgoing links of the given node *i*, SC links are in green and EC links in black. (**a**) Incoming *only SC rate*. (**b**) Outgoing *only SC rate*. (**c**) Incoming *only EC rate*. (**d**) Outgoing *only EC rate*. All ratios were computed at the node level (top 15 entries) and results grouped by functional network and averaged across subjects. Transparent bars refer to the structurally informed DCM results with thresholds of 60%, 40% and 20%, respectively (percentage of kept links). ns = not significant, Wilcoxon signed-rank test with Benjamini/Hochberg multiple comparison correction (H0: symmetric at 0.5), for simplicity highlighted only if non-significant.

For each cortical node, we firstly divided its strongest links (both SC and EC) into within-network and between-network links, and among them we counted the number of *only EC*, *only SC* and *overlap* links. The explanatory cartoon on the top of Fig. 4 focuses on within-network links and it illustrates the example of a node with 2 *only SC* links, 1 *only EC* link and 3 *overlap* links. We used this information to compute the *only SC rate* as described by Eq. (5). Results are shown in Fig. 4,b, and similarly for the *only EC rate* in Eq. (6), reported in Fig. 4c,d. We found that the within-network *only SC rate* clearly changed between LCN and transmodal networks. In detail, LCN has a ratio significantly lower than 0.5, i.e. a small number of *only SC* links compared to the *overlap* links, while transmodal networks have an *only SC* rate closer to 0.5, i.e. a comparable number of *only SC* links and *overlap* links. From the side of the strongest EC links, the *only EC rate* is always smaller than 0.5, meaning that most effective links are supported by a structural link (Fig. 4c,d). Transparent bars refer to the structurally informed DCM results with thresholds of 60%, 40% and 20%, respectively (percentage of strongest kept links). As expected, since the EC was restricted to have non-zero entries only where there were corresponding SC entries, these rates tend to 0 with the percentage of kept links getting lower.

**Figure 5:**
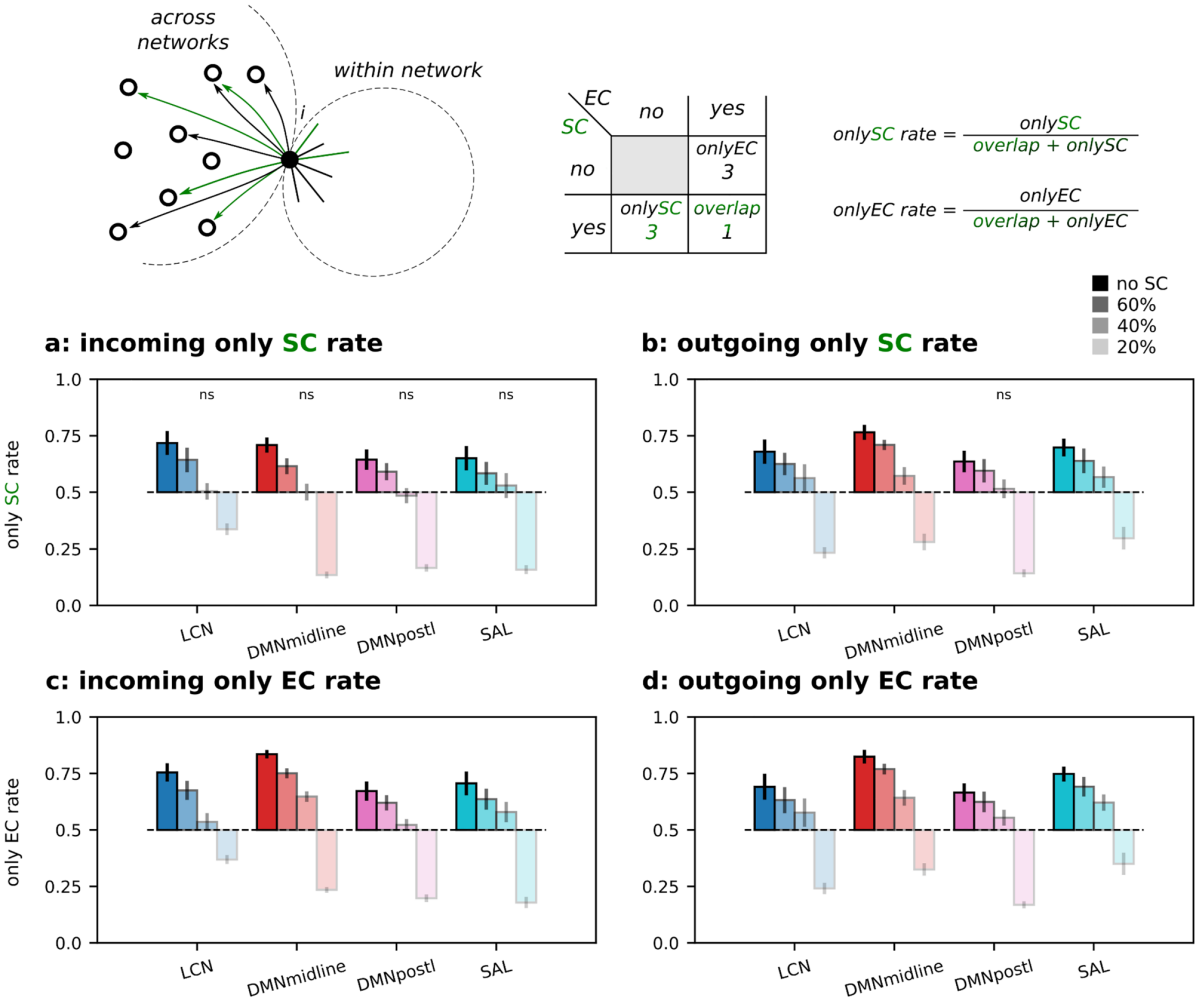
*Only SC* and *only EC* rates across networks. Explanatory cartoon of the *only SC* and *EC* rates computed on the across network outgoing links of the given node *i*, SC links are in green and EC links in black. (**a**) Incoming *only SC rate*. (**b**) Outgoing *only SC rate*. (**c**) Incoming *only EC rate*. (**d**) Outgoing *only EC rate.* All ratios were computed at the node level (top 15 entries) and results grouped by functional network and averaged across subjects. Transparent bars refer to the structurally informed DCM results with thresholds of 60%, 40% and 20%, respectively (percentage of kept links). ns = not significant, Wilcoxon signed-rank test with Benjamini/Hochberg multiple comparison correction (H0: symmetric at 0.5), for simplicity highlighted only if non-significant.

We repeated the analysis by considering across-network links. When using non-structurally informed DCM, both *only SC* and *EC rates* were found to be greater than 0.5 (for both incoming and outgoing links, see Fig. 5a,d full-color bars). This means a low overlap between strong EC and SC links. Moreover, by limiting DCM to the strongest SC links, the overlap increased with the decrease in the percentage of kept links, see Fig. 5a,d transparent color bars. In particular, *only SC/EC rates* dropped below 0.5 when EC was limited to the 20% strongest SC links.

**Figure 6:**
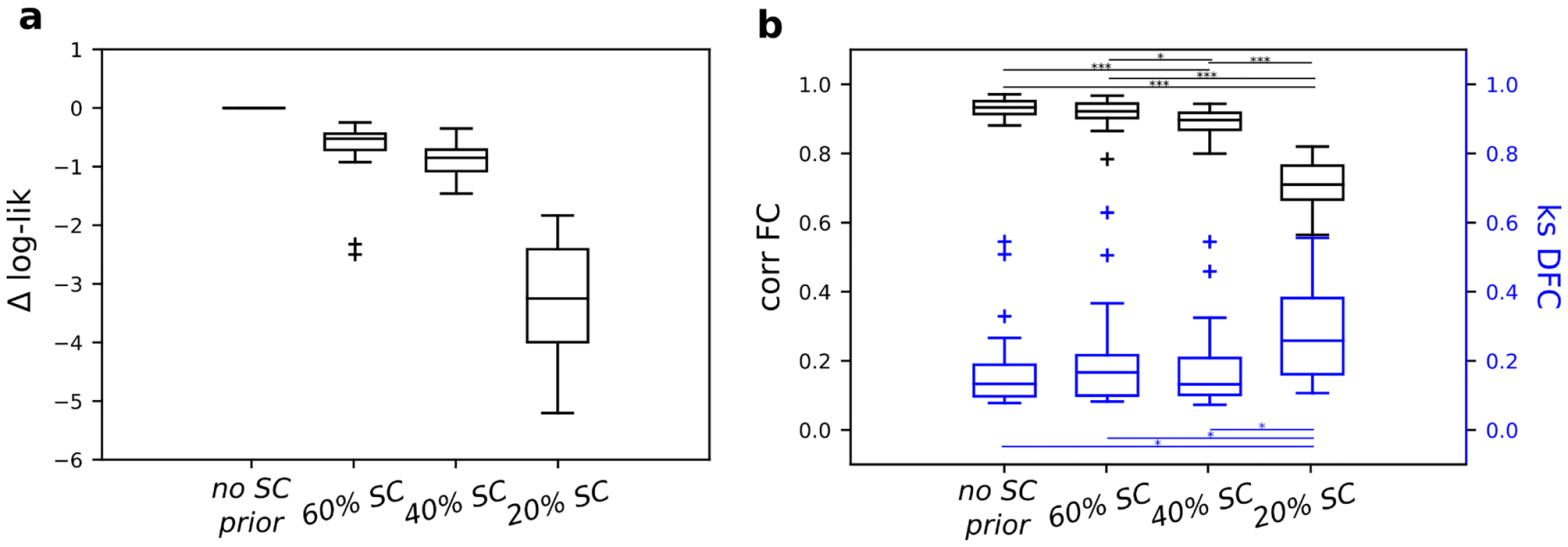
Model fit quantified as: (**a**) the log-likelihood difference between the structurally informed DCMs and the non-structurally informed DCM (used as reference, at the subject level); (**b**) the capability of generating data similar to the empirical recordings used to fit the DCM model, in terms of correlation between their FCs (left y-axis) and KS distance of their dynamic FCs (right y-axis), for non-structurally informed DCM and structurally informed DCM with different SC thresholds, i.e. 60, 40 and 20 (percentage of kept links), averaged over 100 realizations. *p < 0.05, **p < 0.01 and ***p < 0.001, Mann–Whitney U test with Benjamini/Hochberg multiple comparison correction.

The impact of a structural constraint on the goodness of fit to the empirical data is shown in Fig. 6. We computed the variation of log-likelihood for each subject with the non-structurally informed DCM as the upper bound reference (Fig. 6a). On average, as expected it shows a reduction of the log-likelihood if a structural constraint was added. Importantly, the most substantial reduction occurs when transitioning from retaining 40–20% of the links. Furthermore, we used DCM to generate 100 realizations of BOLD data for each subject and an increasing degree of structural constraint. We assessed the correlation between empirical and simulated FC matrices (Fig. 6b, left y-axis), and Kolmogorov–Smirnov distance between dynamic FCs (Fig. 6b, right y-axis). The former deteriorated significantly when transitioning from 60 to 40% of retained links, whereas the latter from 40 to 20%. Collectively, these results indicate that the most stringent structural constraint (20% SC) had the most notable effect on the generative performances of the model, and it also led to the strongest reduction in *only SC/EC rates* (across-networks this is the only case where rates became less than 0.5).

Adding a structural constraint also affected both couplings computed above, i.e. EC–SC and SC–EC couplings. Structurally informed DCM gave the expected functional rank in both couplings. The higher the constraint imposed, the more the functional hierarchy became unmistakable across functional networks, see Fig. S2. Interestingly, this is true even for the SC–EC coupling that did not give the expected unimodal-transmodal hierarchy in the non-structurally informed case, Fig. 3b,c. Moreover, the rank is more pronounced with the incoming coupling in both EC–SC and SC–EC (Fig. S2a/c).

## Discussion

The relation between structural and functional properties of brain connectivity is a central topic in neuroscience. Previous studies have shown the lack of a one-to-one correspondence between structural and functional connectivity. In line with this, a branch of literature has rapidly grown on local structure-functional coupling, showing a strong dependence with cortical hierarchies^14,15,18^. Most of these results were obtained on human data by studying the relation between FC and SC. Here, we employed the mouse brain model for which an accurate reconstruction of the axonal paths^29,41^ and a consolidated protocol to acquire resting-state fMRI data are available^37^. Moreover, the recent developments in the framework of dynamical causal models (DCM) have given variants of DCM suited to compute effective connectivity (EC) in whole-brain networks^22–24^. In the context of DCM, the advantage given by a structurally informed prior has been largely proved by tuning the prior variance of each effective link proportionally to the likelihood that such link anatomically exists^8,9^.

Here, we started with a non-structurally informed DCM and compared each single mouse EC with the mouse structural connectome obtained at the population level. We found that a higher mean structural strength corresponds to a higher mean effective strength, and vice versa; this is consistent with prior studies showing how DCM benefits from structural information^8,9^. However, our aim was to further our understanding of coupling between EC and SC at the node level, and characterize their overlap. Importantly, we found that EC–SC coupling, i.e. the coupling driven by the strongest effective links, follows the unimodal-transmodal functional hierarchy at the cortical level previously identified in the mouse^29^. This relation replicates and expands human findings, by showing that the relationship previously observed in humans is largely driven by EC–SC, and not vice versa^42–44^. In this respect, the coupling of the salience (SAL) network is of particular interest, as both the incoming and outgoing connections of SAL have the lowest coupling strength compared to the other functional networks. This is in line with what was previously reported in Liu et al.^18^, where the authors found that SAL is one of the most dynamic networks in terms of structure-functional coupling.

Interestingly, SC–EC coupling (i.e. the coupling driven by the strongest structural links) did not replicate the same cortical hierarchy found with EC–SC, meaning that when conditioning on the structure, links that do not meet the expected relationship were included. This prompted us to focus on the overlap between EC and SC. Our results revealed two different scenarios, depending on whether we considered within or between network links. Specifically, within high-order cortical areas were found to have strong structural connections that did not exhibit a correspondingly strong effective link. The mismatch between SC and EC was even more clear on links across networks, as most strong effective links were not supported by a structural one. Only connections within unimodal sensory motor networks aligned both in terms of effective and structural strength, highlighting the different degree of segregation of each network. This finding is consistent with the fact that the unimodal somatomotor network is densely connected and spatially close, while this is not the case for the DMN or higher-level transmodal networks^1,45^.

A different perspective to look at the heterogeneity of structural and functional connections considers how communication occurs in the network^46^. The specific organization of links within and between networks has been recently explained by considering the preferred pattern of structural connections that each network adopts to communicate^16,47^. By rephrasing our results in these terms, the stronger structure-functional coupling in unimodal regions is a consequence of the preferred local scale, i.e. monosynaptic links, on which communication occurs. Moving along the hierarchy toward the transmodal cortex, the optimal scale globally extends involving more polysynaptic pathways. Therefore, our observations of a different connection profile across unimodal and polymodal networks in terms of SC and EC might be explained with a gradient of optimal scales ranging from local (within LCN, where SC and EC overlap), to a more global scale occurring between networks. Additional evidence on the effect of monosynaptic vs. polysynaptic paths is that the SC–EC coupling significantly decreased in the incoming links of some subcortical nodes, i.e. lateral septal complex, pallidum, thalamus and hypothalamus. This finding is consistent with previous observation of a higher mismatch between FC and SC in subcortical networks due to the polysynaptic nature of their anatomical links^28^. A connection to the reduction in incoming SC–EC coupling of subcortical areas can also be drawn from the findings reported in Gutierrez-Barragan et al.^34^. In this study, the anesthetized cohort, specifically under halothane anesthesia, exhibited a lower functional connectivity between subcortical regions, particularly the basal forebrain and hypothalamus, and cortical areas compared to the awake cohort. This observation was justified by recognizing the pivotal role of the involved areas in mediating arousal and vigilance in the mammalian brain. Considering the significance of information integration in consciousness^48^ and the central role of the thalamus in integrating cortical information^37^, the effects of anesthesia may provide an explanation for the reduced incoming SC–EC coupling in subcortical areas. However, it is important to note that there is substantial prior evidence supporting the suitability of the adopted anesthesia protocol for measuring rsfMRI data. Notably, studies such as^45,49^, have reported the preservation of functional networks akin to those observed in conscious rats and primates under this regime of light anesthesia (halothane, 0.75%). Furthermore, this anesthesia protocol has been found to maintain the characteristic spectral properties of the BOLD signal, as indicated in Gutierrez-Barragan et al.^34^.

In an attempt to better understand the coupling between SC and EC, we also included a structural constraint on sparse-DCM. Specifically, we forced EC to be always supported by a structural link and we repeated the inference for different percentiles of kept structural connections. Following the previous interpretation of multiscale communication, by constraining EC to SC, we limited the spectrum of the spatial scale to be more local as the percentage of kept links decreased. This analysis yielded three results: firstly, as expected the overlap between strong EC and SC links was found to be increased, i.e. the *only SC* and *EC* ratios as shown in Figs. 4 and 5, tended to zero both within and across networks. Secondly, when strongly constrained by the structure, the previously observed functional hierarchy became more evident (Supplementary Fig. S2) both when computed through EC–SC coupling and SC–EC coupling. Incoming effective links revealed a well distinct cortical rank that reflects the expected hierarchy. This was mainly due to LCN nodes in which their effective links strongly coupled with their structural ones. This finding is in line with the results in Sokolov et al.^50^, where the authors showed that DCM with a prior built on incoming structural information outperformed models informed by outgoing structural information as well as those without structural information. Lastly, the third finding relates to the fact that DCM is a generative model, as a result, it can produce new realizations of the same dynamical system. Within this framework, we tested how the capability of our model to generate data as similar as possible to the empirical ones, varied in relation to a structural constraint. Our results showed a decrease in model performance (both in terms of fit to the empirical signals -which was expected as the structural constraint limited the parameter space- and capability to reproduce the empirical static and dynamic functional connectivity) when the structural constraint was too strict. Here, it is important to note how the overlap between SC and EC got higher when the structural constraint became stricter in DCM, in line with^47^. Altogether, these results suggest that a structural constraint on DCM should consider the heterogeneity of the EC/SC coupling: it can be more severe on links within sensorimotor network, whereas a higher degree of freedom might be needed especially on across-network links.

The interpretation of these results must also take into account some limitations. First, while using the murine model, we leveraged the possibility of differentiating incoming and outgoing SC connections. However, it is unclear how well these results apply to higher mammalian species characterized by larger white matter tracts, and by proportionally denser long-range connectivity^51^. Second, by using the mouse brain connectome, i.e. a single structural matrix, the inter-subject variability could not be considered, while recent studies have demonstrated that interindividual variability in mouse^52^ and in human^44^, along with other factors, e.g. time dependence^18^, and receptor maps^53^, play a critical role in relating structure to function. This could have potentially resulted in an overestimation of the percentage of retained links that corresponded to a significant deterioration in model performance (Fig. 6b).

In summary, the present work studies the relation between SC and EC in terms of their coupling and overlap by conditioning on the strongest SC and EC links. The novelty of our work concerns mainly three aspects: (1) it brings EC into the field of structure–function coupling, (2) it differentiates between EC–SC and SC–EC couplings (by conditioning on the strongest EC and SC links, respectively) exploring how non-structurally informed EC and SC differ, and (3) it characterizes the effect of structurally constraining DCM in terms of both the relation between SC and EC and the generative capability of the model. Briefly, we found that when conditioning on the strongest EC links, the coupling follows the unimodal-transmodal functional hierarchy. Whereas the reverse is not true, indeed there are strong SC links within high-order cortical areas which do not correspond to a strong EC link. This mismatch is even more clear across networks. Only connections within sensory motor networks freely align both in terms of effective and structural strength.

### Animal experimental procedure

A dataset of n = 20 adult male C57BI6/J mouse resting-state fMRI recordings were previously acquired at the IIT laboratory (Italy). All in vivo experiments were conducted in accordance with the Italian law (DL 2006/2014, EU 63/2010, Ministero della Sanità, Roma) and the recommendations in the Guide for the Care and Use of Laboratory Animals of the National Institutes of Health. Animal research protocols were reviewed and consented by the animal care committee of the Italian Institute of Technology and Italian Ministry of Health.

### Supplementary Information

Supplementary Information.

## Data Availability

The data supporting the findings of this study are available in the following GitHub repository: https://github.com/danilobenozzo/sc_ec_coupling_mouse.git The mouse rs-fMRI recordings are available from the corresponding author on reasonable request.
