# Supplementary material for: Macroscale coupling between structural and effective connectivity in the mouse brain
Source: Sci Rep. 2024 Feb 7;14:3142. doi: 10.1038/s41598-024-51613-7 (PMC10850485; doi:10.1038/s41598-024-51613-7)
Supplement: Supplementary file 1 — Supplementary Information. [file 41598_2024_51613_MOESM1_ESM.pdf]

Supplementary Materials

MO	Motor area	primary (MOp), secondary (MOs)
SS	Somatosensory area	primary (SSp), supplementary (SSs)
GU	Gustatory area	
VIS	Visual area	
AUD	Auditory area	
ACA	Anterior cingulate area	dorsal (ACAd), ventral (ACAv)
PL	Prelimbic area	
ILA	Infralimbic area	
ORB	Orbital area	
AI	Agranular insular area	dorsal (AId), posterior (AIp), ventral (AIv)
RSP	Retrosplenial area	agranular (RSPagl), dorsal (RSPd), ventral (RSPv)
PTLp	Posterior parietal association areas	
TEa	Temporal association areas	
PERI	Perirhinal area	
ECT	Ectorhinal area	
VISC	Visceral area	
PIR	Piriform area	
HPF	Hippocampus	Ammons's horn (CA), Dentate gyrus (DG), Entorhinal area (ENT), Subiculum (SUB)
CTXsp	Cortical subplate	
STR	Striatum region	dorsal (STRd), ventral (STRv), Lateral Septal Complex (LSX), striatum-like Amygdalar Nuclei (sAMY)
PAL	Pallidum	
THAL/HY	Thalamus/Hypothalamus	sensory-motor cortex related (DORsm), polymodal association cortex related (pmDOR)

Table ST1. ROIs name and abbreviations, third column specifies the internal subdivisions of the adopted parcelization.

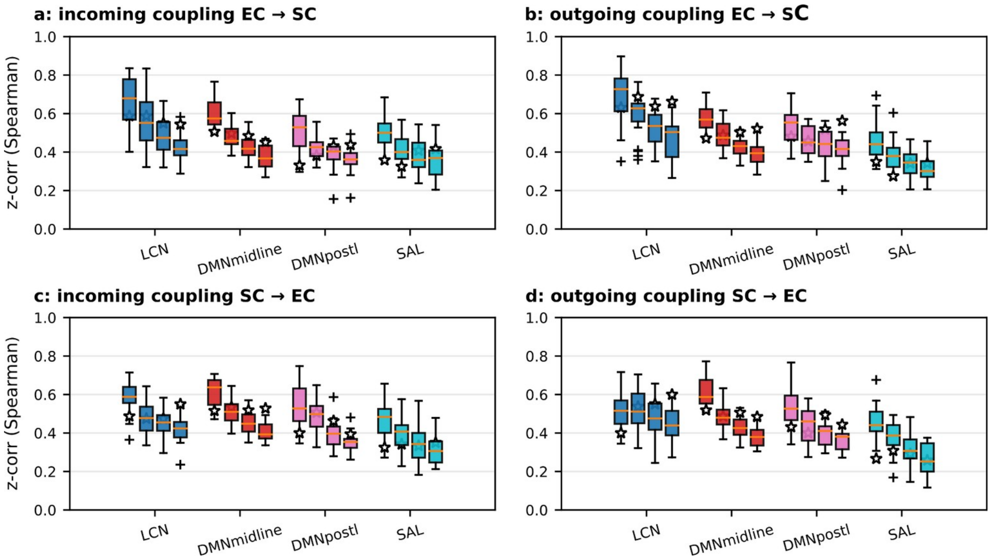

Figure S1. (a-b) EC-SC coupling, an expanded version of Figure 2b-c across different values of threshold k (k=15, 20, 25, 30) that is the number of top EC incoming and outgoing entries selected in each node, on which the Spearman rank correlation was computed with the corresponding SC entries. (c-d) SC-EC coupling, similar to Figure 3b-c, extended by increasing the threshold k from 15 to 30 (step 5), as before. The first boxplot in each quartet refers to k=15, thus equals to what reported in the main text. In each boxplot, white start marker shows the average rate of nodes with significant correlation in that functional network. Significance was computed

by random permutations test (p=0.05, n=500). Overall, it shows a decrease in the coupling when k increases, and also a higher EC-SC coupling in LCN which makes the gradient from unimodal to transmodal networks more evident.

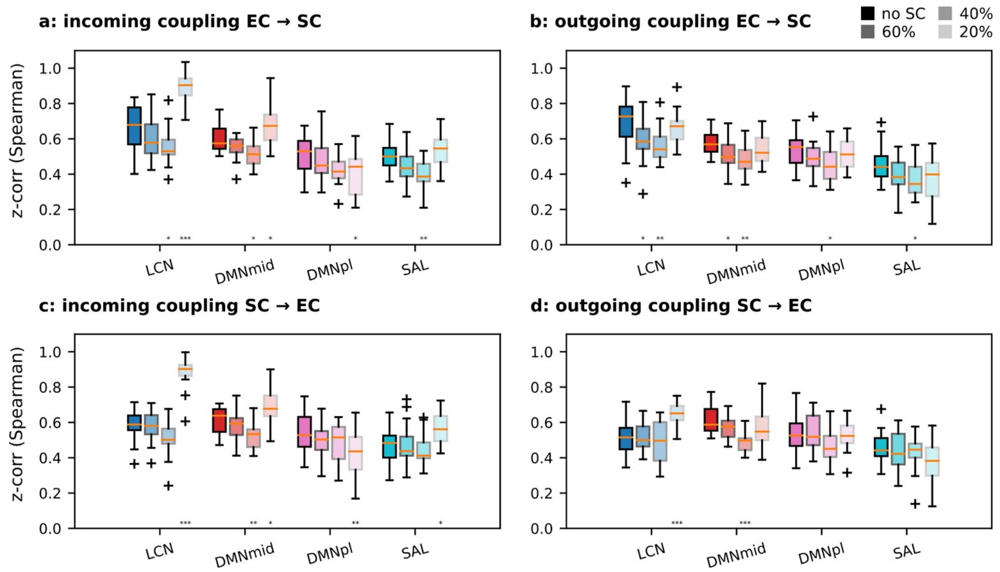

Figure S2. (a-b) EC-SC coupling, similar to Figure 2b-c, extended with the couplings computed on the results from the structurally-informed DCM with different SC thresholds, i.e. 60, 40 and 20% (percentage of kept links proportional to the level of transparency of the boxplots). (c-d) SC-EC coupling, similar to Figure 3b-c, extended with the structurally informed results, as before. *=p<0.05, **=p<0.01 and ***=p<0.001, ANOVA test within network, reported significance against the non-structurally-informed DCM, with Tukey's multiple comparison test.

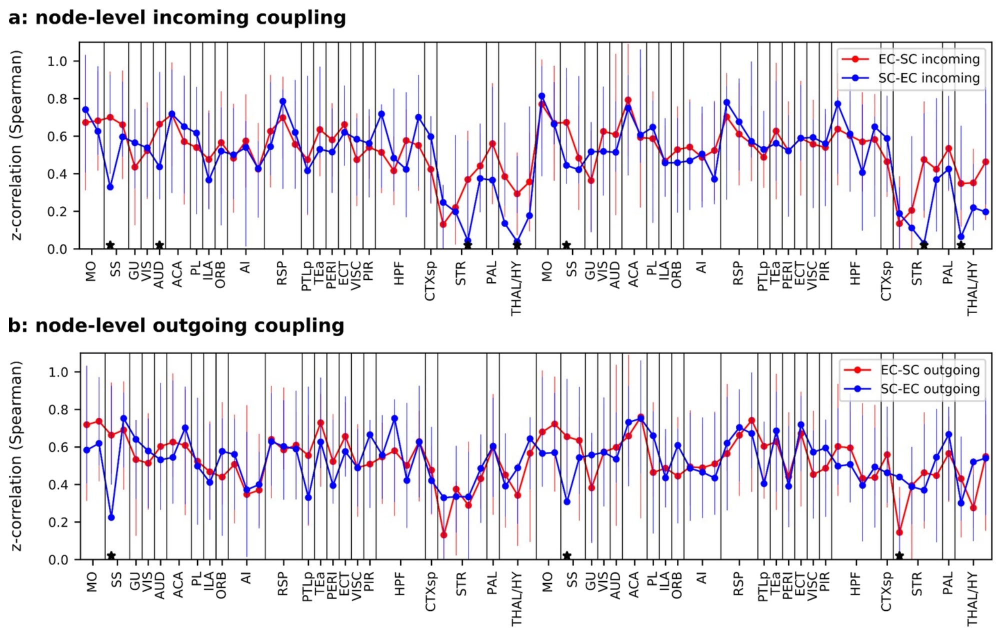

Figure S3. Node-level coupling separately of incoming (a) and outgoing links (b) averaged across subjects. Each panel shows the related EC-SC (red) and SC-EC (blue) couplings computed as the Spearman rank correlation between the strongest k=15 entries of EC and SC, respectively, and the corresponding counterpart in SC and EC. *=p<0.05, paired t-test, Benjamini/Hochberg multiple testing correction. Of interest is the primary somatosensory area (SSp, the first parcel of SS in the figure), which exhibits significantly lower SC-EC coupling for both incoming and outgoing links. This observation is reminiscent of what reported in Grandjean et al., 2017 (see the top-left panel of Fig.2), where a seed-based FC analysis revealed stronger contralateral connections of the SSp then what was indicated by the SC. From our analysis, it results that 87% (13/15) of the strongest incoming SC connections of SSp and 73% (11/15) of its strongest outgoing connections are ipsilateral. In contrast, when examining EC connections of SSp, the percentages decrease to 49% (7.4/15, average across subjects) for the incoming links, and to 57% (8.5/15, average across subjects) of the outgoing links. These discrepancies might explain the reduced coupling when the selection of links was guided by SC.

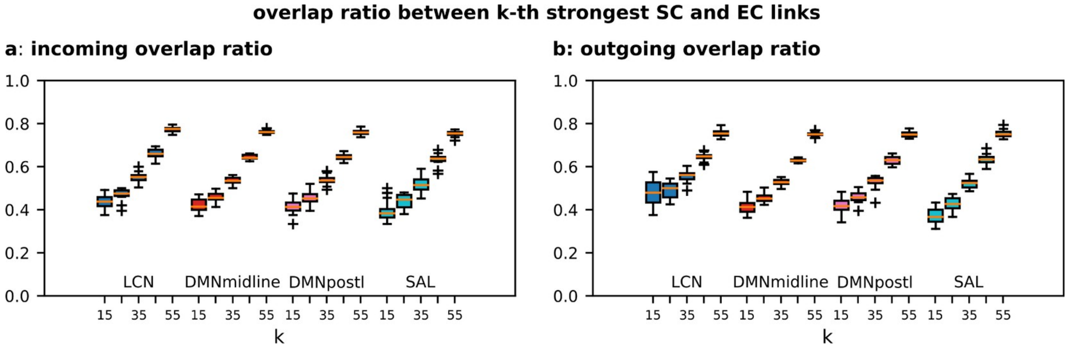

Figure S4. Overlap ratio between k-th strongest SC and EC links computed on each node and grouped by functional networks, k from 15 to 55 (step 10).
